# Supplementary material for: Correction: Complex Environmental Forcing across the Biogeographical Range of Coral Populations
Source: PLoS One. 2015 Apr 16;10(4):e0126756. doi: 10.1371/journal.pone.0126756 (PMC4400167; doi:10.1371/journal.pone.0126756)
Supplement: S1 File — (PDF) [file pone.0126756.s001.pdf]

PLOS ONE

RESEARCH ARTICLE

Complex Environmental Forcing across the Biogeographical Range of Coral Populations

Emily B. Rivest[1] *, **Tarik C. Gouhier**[2]

1 Bodega Marine Laboratory, University of California Davis, Bodega Bay, California, United States of America, 2 Marine Science Center, Northeastern University, Nahant, Massachusetts, United States of America

* ebrivest@ucdavis.edu

Abstract

Although there is a substantial body of work on how temperature shapes coastal marine ecosystems, the spatiotemporal variability of seawater pH and corresponding *in situ* biological responses remain largely unknown across biogeographic ranges of tropical coral species. Environmental variability is important to characterize because it can amplify or dampen the biological consequences of global change, depending on the functional relationship between mean temperature or pH and organismal traits. Here, we characterize the spatiotemporal variability of pH, temperature, and salinity at fringing reefs in Moorea, French Polynesia and Nanwan Bay, Taiwan using advanced time series analysis, including wavelet analysis, and infer their potential impact on the persistence and stability of coral populations. Our results demonstrate that both the mean and variance of pH and temperature differed significantly between sites in Moorea and Taiwan. Seawater temperature at the Moorea site passed the local bleaching threshold several times within the ~45 day deployment while aragonite saturation state at the Taiwan site was often below commonly observed levels for coral reefs. Our results showcase how a better understanding of the differences in environmental conditions between sites can (1) provide an important frame of reference for designing laboratory experiments to study the effects of environmental variability, (2) identify the proximity of current environmental conditions to predicted biological thresholds for the coral reef, and (3) help predict when the temporal variability and mean of environmental conditions will interact synergistically or antagonistically to alter the abundance and stability of marine populations experiencing climate change.

OPEN ACCESS

Citation: Rivest EB, Gouhier TC (2015) Complex Environmental Forcing across the Biogeographical Range of Coral Populations. PLoS ONE 10(3): e0121742. doi:10.1371/journal.pone.0121742

Academic Editor: Rodolfo Paranhos, Instituto de Biologia, BRAZIL

Received: October 3, 2014

Accepted: February 15, 2015

Published: March 23, 2015

Copyright: © 2015 Rivest, Gouhier. This is an open access article distributed under the terms of the Creative Commons Attribution License, which permits unrestricted use, distribution, and reproduction in any medium, provided the original author and source are credited.

Data Availability Statement: All environmental data files are available from the LTER Metacat data catalog, mirrored in DataOne (accession number knb-lter-mcr.5014).

Funding: This study was funded by the Moorea Coral Reef Long-Term Ecological Research Program (http://mcr.lternet.edu), the National Science Foundation (OCE-1220359 to Gretchen Hofmann), and the National Museum of Marine Biology and Aquarium in Taiwan (http://www.nmmba.gov.tw). EBR was funded by the National Science Foundation Graduate Research Fellowship (http://www.nsf.gov/grfp) and the Doctoral Dissertation Enhancement Program (http://www.nsf.gov/publications/pub_summ.

Introduction

Efforts to describe natural environmental dynamics in different marine near-shore environments have greatly enhanced the study of global ocean change, specifically ocean warming and ocean acidification (OA; the decline in seawater pH and carbonate ion concentration due to absorption of anthropogenic CO_2 by the ocean). Recently, the challenge of acquiring high-frequency, long, continuous environmental pH datasets that estimate these changing

jsp?ods_key = nsf04036&org=NSF). TCG was funded by the National Science Foundation (CCF-1442728).

Competing Interests: The authors have declared that no competing interests exist.

conditions for populations of benthic species has been overcome with the advent of autonomous oceanographic pH sensors, such as those called SeaFETs [1]. Deployed and tested in sites ranging from tropical to polar, these sensors have shown that model predictions of global ocean pH underestimate the natural variation in seawater pH occurring between marine ecosystems [2–5]. With these sensors, the research community can now collect high-frequency environmental data to complement IPCC projections and to provide details of the conditions that adults and larvae experience *in situ* [6].

Data from autonomous pH sensors have shifted scientific consensus away from the idea that OA is a homogenous, global environmental stressor. The acidification of the local ocean and its effects may manifest in site-specific ways because not all populations of the same species experience the same variability of present-day environmental conditions. For example, SeaFETs deployed within the Moorea Coral Reef and Santa Barbara Coastal Long-Term Ecological Research (LTER) sites have documented marked differences in pH regimes across a variety of spatial scales [7]. pH variability through time differed between kelp forest habitats in the Santa Barbara channel only 54 km apart, despite similar species diversity and mean pH values at both sites. In Moorea, SeaFET sensors documented different pH variability across a transect of the barrier reef, a spatial scale of ~1 km. In Hawaii, pH variability differs across a spatial scale of 35 m from shore through a fringing reef [8]. These data highlight the spatially complex mosaic of pH conditions that a study population may experience. Differences in pH variability across these small spatial scales (1–50 km) may foster a diversity of pH-related phenotypes within or among breeding populations, increasing the potential for resilience of species to future changes in environmental pH at regional scales. Additionally, the deployments of pH sensors provide an environmental context for interpreting the results of laboratory experiments where organisms are exposed to pCO_2 treatments. The LTER SeaFET deployments, among others, highlight the need to co-locate pH and other autonomous sensors with biological experiments as a holistic approach to studying global change biology in coastal oceans [4,7,9].

Studies regarding the variability of pH and *in situ* biological response for coastal marine ecosystems are accumulating (*e.g.*, [3,8,10–15]). A few studies have linked natural variability of carbonate chemistry on coral reefs with community-level performance. Price *et al.* [4] and Albright *et al.* [16] have shown that *in situ* pH variability correlates with community succession and rates of net accretion and net production on coral reefs. Lower daily pH causes slower net accretion and results in greater relative abundance of non-calcifying benthic taxa [4]. Shaw *et al.* [17] reported community calcification rates across a wide range of carbonate chemistry, predicting future declines in accretion under OA. More recently, several studies have paired these types of physical measurements with direct measurements of biological response of resident organisms to future ocean conditions [6,9,17–21]. Yu *et al.* [6] found that sea urchins in the Santa Barbara Channel experience high fluctuations of pH due to seasonal upwelling (about 8.2–7.7). When larvae from this population were raised under pH 7.7 and pH 7.5, they developed normally and showed similar morphological and growth responses. These results suggested that populations of sea urchins that naturally experience high variation of ambient pH may be more resilient to anthropogenic changes in acidity, though no comparison with a population of low-variability environmental pH was conducted. Additionally, Kelly *et al.* [19] found evidence for local adaptation to carbonate chemistry conditions in sea urchin populations on the Oregon coast. From these studies, we are beginning to appreciate how regional scale variation might influence biological responses to long-term environmental change as well as the adaptive potential of populations [6,22–26].

While populations located in geographical areas of active climate-mediated change in pH are often characterized as vulnerable, these populations could also serve as genetic reservoirs, showing adaptation or acclimatization to otherwise stressful pH conditions. In general, tropical

reef corals may be locally acclimatized or adapted to the pH and temperature variation under which their populations have evolved, a process that is well known in terms of local adaptation to temperature in marine invertebrates (reviewed in [27]). Coral populations that have experienced high variability in pH or frequent pulses of low pH seawater, for example from upwelling, may have developed the physiological capacity to tolerate the global average low pH, high pCO_2 conditions predicted to occur by the end of this century. Physiological adaptation to historical environmental conditions has been shown with Sockeye salmon in British Colombia, Canada. Population-specific cardiorespiratory performance in these fish is optimized to the temperatures experienced during annual river migrations [28]. Similarly, though they are likely reproductively isolated, groups of coral at locations of differing pH conditions may have varying degrees of physiological plasticity reflecting the historical nature of their carbonate chemistry environment.

Under a scenario of adaptation and acclimatization [26], understanding the spatiotemporal variability of present-day environmental conditions is important for (i) predicting the stability and resilience of marine populations experiencing climate change and (ii) devising successful management and mitigation strategies for these populations. Current efforts are largely focused on detecting geographical areas of rapid and more gradual climate-mediated change in mean pH in order to identify potentially vulnerable populations. While identifying such spatial differences in mean pH conditions is a critical first step, it is equally important to recognize that temporal variation in pH conditions can either amplify or dampen the effects of mean pH on marine organisms [29,30]. Indeed, Jensen's inequality, a mathematical consequence of averaging nonlinear functions, suggests that the effect of the variance of pH will depend on the (nonlinear) functional relationship between mean species abundance and mean pH conditions. If mean abundance is an accelerating (decelerating) function of mean pH, then temporal variance in pH will increase (reduce) mean abundance [29]. Hence, the temporal variance and the mean of pH can either interact synergistically or antagonistically to affect the stability and persistence of marine populations. Consequently, to accurately assess the vulnerability of marine populations, managers should take into account two components of pH, namely spatial heterogeneity in mean pH and local temporal variability in pH. Several studies using autonomous sensors have successfully used variability of environmental pH as environmental context for measurements of biological performance and ecosystem processes [4,9,20,21,31,32]. These types of studies, including the work presented here, serve to ground our understanding of the future biological and ecological effects of OA by providing a frame of reference of the present-day regime experienced by the study population as well as a better understanding of the spatial and temporal variability in conditions and responses through which the effects of changing ocean conditions on marine ecosystems will manifest.

In this study, we compared environmental conditions between fringing reef locations at Moorea, French Polynesia and Nanwan Bay, Taiwan and inferred their potential impacts on the stability and persistence of local coral populations. We predicted that pH and temperature would differ between reef locations because previous work has shown that Nanwan Bay experiences tidally-driven upwelling events [33], while the fringing reef in Moorea is more protected inside a lagoon. In addition, this geographic comparison (Moorea-Taiwan) would likely include a difference in total alkalinity, perhaps by 100 μmol kg SW^{-1} (*e.g.*, [34]).

Materials and Methods

Environmental data collection in Moorea

From January 20 to March 16, 2012, time series of pH, temperature, and depth were recorded for a fringing reef on the north shore of Moorea (17.4803 S 149.7989 W). All fieldwork at this

location was performed under an annual research permit issued by the French Polynesian Ministry of Research to EBR. Moorea, French Polynesia is a volcanic island within the Society Island archipelago in the south-central Pacific Ocean. The island is surrounded by a barrier coral reef, which protects an inshore lagoon as well as a fringing reef. There are several passes in the barrier reef that allow seawater exchange between the lagoon and the open ocean. The study site was located on the fringing reef on the north shore of Moorea, approximately 500 m alongshore from a pass through the barrier reef. Seasonal climate in Moorea is characterized by dry (May to October) and rainy (November to April) seasons. Winds can contribute to seawater circulation patterns on the North shore, where easterly trade winds are common. During the wet season, prevailing winds come from the north-east; during the dry season, the island shades the North shore from the prevailing southeasterly tradewinds.

pH was recorded continuously using an autonomous data logger based on a Honeywell Durafet pH sensor, called a SeaFET [1]. The SeaFET was deployed at 3.3 m depth and suspended approximately 0.6 m off the sandy bottom; the instrument measured pH voltage at 10-minute intervals, averaging data over 30-second periods. The deployment location was approximately 33 m from the collecting location of adult *Pocillopora damicornis* used in laboratory experiments [32]. Adjacent to the SeaFET were two thermisters (SBE 39, Sea-Bird Electronics, Bellevue, WA) and two HOBO water level data loggers (U20, ONSET Computer Corp., Bourne, MA), synchronized with the SeaFET to simultaneously record temperature and depth. SeaFET electrode surfaces and depth loggers were caged in copper mesh to deter fouling; cages were cleaned twice a month with a toothbrush when the sensor was idle between measurements.

Following deployment, the SeaFET electrodes were allowed to stabilize and then were calibrated using discrete seawater samples collected *in situ*, a method based on ISFET electrode characteristics [1]. On February 16, 2012, a SCUBA diver using a Niskin bottle collected a single calibration sample adjacent to the SeaFET concurrently with its voltage reading. Temperature of the seawater *in situ* was measured using an alcohol thermometer. The seawater sample was used to rinse and fill two 500 mL borosilicate glass bottles with minimal contact of seawater and atmospheric air. The bottles were sealed with gas-impermeable grease and a ground-glass stopper [35]. Analyses of carbonate chemistry were performed within 1–2 hours of sample collection.

Environmental data collection in Taiwan

The study site in Taiwan is a fringing reef in Nanwan Bay, located at the southern tip of the island. Nanwan Bay, Taiwan is a semi-enclosed basin, 14 km across, and bounded by two capes with the Pacific Ocean to the east and the Taiwan Strait to the west. Fringing reefs and sandy beaches line the coast, with several seamounts in the middle of the bay. On the east side of the bay, near Hobihu and the study site, there is no shallow continental shelf. Circulation patterns in Nanwan Bay are driven by tides, with added influence of high winds during monsoon/typhoon season (June-October).

From May 24 to July 12, 2012, time series of pH, temperature, salinity, and depth were recorded for a fringing reef in Nanwan Bay, Taiwan (21.9385 N 120.7967 E). pH was recorded continuously using a SeaFET [1], which was deployed at ~4 m depth and suspended approximately 0.6 m off the sandy bottom. The instrument measured pH voltage at 6-minute intervals, averaging data over 30-second periods. Adjacent to the SeaFET were two conductivity/temperature sensors (SBE 37, Sea-Bird Electronics, Bellevue, WA) and two HOBO water level data loggers (U20, ONSET Computer Corp., Bourne, MA), synchronized with the SeaFET to simultaneously record conductivity, temperature, and depth. Fouling of SeaFET electrode surfaces and depth loggers was deterred as described above. The deployment location for the sensors

was located within 33 m of the collecting locations of adult *P. damicornis* used in concurrent laboratory experiments (data not described here). No permit was required to deploy the oceanographic sensors at this location.

Following deployment, discrete seawater samples were collected adjacent to the electrodes in the SeaFET sensor were allowed to stabilize and then were calibrated using discrete seawater samples collected *in situ* [1] on July 2, 2012, following procedures described above.

Seawater chemistry analyses of SeaFET calibration samples

pH, total alkalinity (A_T), and salinity from bottle samples were measured in four replicates within 1–2 hours of sample collection. Samples in Moorea were analyzed in collaboration with the Moorea Coral Reef Long Term Ecological Research facility at UC Berkeley's Richard B. Gump South Pacific Research Station. Samples in Taiwan were analyzed in collaboration with Dr. PJ Edmunds (CSUN) and Dr. T-Y Fan at the National Museum of Marine Biology and Aquarium.

Seawater salinity was measured using a conductivity meter (Moorea: YSI 3100, YSI Inc., Yellow Springs, OH, USA; Taiwan: 340i, WTW GmbH, Weilheim, Germany). Seawater pH was measured using a spectrophotometric method with indicator dye, *m*-cresol purple (SOP 6b, [35]). Following this method, we estimate our accuracy to be -0.030 ± 0.006 pH units. Total alkalinity (A_T) was measured using an automated, closed-cell potentiometric titration (SOP 3b, [35]) using an automatic titrator (Moorea: T50 with DG115-SC pH probe, Mettler Toledo, LLC., Toledo, OH, USA; Taiwan: DL50 with DG101-SC pH probe, Mettler Toledo, LLC., Toledo, OH, USA). Titrations were performed using certified acid titrant (~0.1M HCl, 0.6M NaCl; A. Dickson Laboratory, Scripps Institute of Oceanography), and A_T was calculated following Dickson *et al.* [35]. Analyzed certified reference materials from A. Dickson Laboratory were titrated to confirm the precision and accuracy of the process; these reference titrations were accurate within 10 μmol kg SW^{-1} (0.1–0.3%).

pH measured at 25°C was converted to pH at environmental temperature using A_T, temperature, and salinity of the bottle samples via CO2CALC [36], with CO_2 constants K1, K2 from [37] refit by [38] and pH expressed on the total scale (mol kg-SW^{-1}). The calculated bottle pH at environmental temperature and salinity was then compared with the voltage recorded by the sensor at the time points of sample collection to determine the value E^* for the Nernst equation. This equation was then used to calculate pH from raw sensor voltage for the entire time series [1]. In Moorea, the salinity value measured from the bottle sample was assumed constant and used for the entire time series. In Taiwan, the salinity time series was used for this calculation. All salinity values here are presented as a unitless quantity representing kg dissolved solids per kg seawater, the standard for oceanography.

After *in situ* pH was calculated, CO2CALC [36] was used to estimate the remaining carbonate chemistry parameters, with CO_2 constants K1, K2 from [37] refit by [38] and pH expressed on the total scale (mol kg-SW^{-1}). Specifically, pCO_2 and the saturation states for calcium carbonate minerals aragonite and calcite (Ω_{arag}, Ω_{calc}) are presented here. For this calculation, constant A_T was assumed for the deployment; average A_T values measured from the bottle samples were used. Assumptions of constant A_T during both 1–2 month deployments were necessary because this parameter could only be measured using discrete samples. Although unavailable for the Taiwan site, previous observations at the Moorea site [32] allowed us to estimate the full range of alkalinity (± 50 μmol kg SW^{-1}). Based on this estimate, we calculated the uncertainty in several other carbonate chemistry parameters for each sample in the time series. The uncertainty in alkalinity results in consequent uncertainties (mean ± SEM) of 10.54 ± 0.02 μatm pCO_2 and 0.0791 ± 0.0001 units of Ω_{arag}, at environmental temperature and

salinity of 35.65. In Taiwan, equivalent uncertainty of alkalinity results in uncertainties (mean ± SEM) of 11.13 ± 0.02 μatm pCO_2 and 0.0723 ± 0.0001 units of Ω_{arag}, at environmental temperature and salinity. For the Moorea data set, salinity was also assumed constant, and the bottle sample average was used for CO2CALC calculations. Based on previous observations at the Moorea site [32], the full range of salinity was estimated to be ± 1. Consequently, there were uncertainties (mean ± SEM) of 3.998 ± 0.007 μatm pCO_2 and 0.0319 ± <0.0001 units of Ω_{arag}, at environmental temperature and 2353 μmol kg SW^{-1}. For the Taiwan data set, the salinity time series was used for CO2CALC calculations. All uncertainty values were calculated for average carbonate chemistry conditions at each site.

Data analysis

All analyses were carried out using MATLAB (v. 7.2.0; Mathworks, Inc.) and R (v. 3.0.1; R Core Team, 2013). First, time series of pH, temperature, salinity, and depth were processed through a series of quality control procedures. Briefly, the time series were trimmed and cleaned to exclude erroneous values. Due to the fact that there was only one calibration sample per time series, drift in SeaFET pH due to biofouling or internal sensor drift was not identifiable. The time series pH, temperature, salinity, and depth were then processed using a one-hour low-pass filter.

We used wavelet analysis to understand how the relative contribution of each frequency or period to the overall variance of the pH, temperature, salinity and depth time series changed over the duration of our surveys [39,40]. Such methods are preferable to more traditional spectral analyses when time series exhibit clear signs of non-stationarity (*i.e.*, the statistical properties of the time series varied over time) [40]. In our case, the time- and frequency-resolved wavelet analyses were particularly useful in resolving the fine-scale fluctuations in pH and temperature. We provide a detailed description of the wavelet analyses in S1 Text and a summary below. We used the Morlet wavelet to decompose the total variance of each signal (*i.e.*, pH, temperature, salinity, depth) over the time and frequency domains. For each signal, this mathematical decomposition yielded a wavelet power spectrum, which was plotted using heatmaps with time on the x-axis, period (which is inversely related to frequency or scales) on the y-axis, and variance on the z-axis. Instances of significantly high temporal variation in the wavelet power spectrum were identified by comparing the observed patterns of variation across the heatmap (*i.e.*, time and periods) to those expected from a null model. The null model was generated by a first order autoregressive process whose parameters were estimated from the observed data [41]. Then, the wavelet power spectrum was either averaged across all periods to produce a scale-averaged time series of power or averaged across all time points to produce the time-averaged power at each period known as the global wavelet power spectrum. The scale-averaged power time series thus represents the average variation of the signal across all periods at each time point whereas the global wavelet power spectrum represents the average variation of the signal across all time points at each period.

Finally, we computed (partial) wavelet coherence to quantify the time- and frequency-resolved local correlation between pH, temperature and salinity [40–42]. This was done by first obtaining the wavelet power spectrum of each time series as described above. Then, we computed the cross-wavelet power spectrum between pairs of time series, which represents their local covariance in the time and frequency domains. To produce wavelet coherence, the cross-wavelet power was then normalized by the product of the wavelet power spectrum of each time series. Thought of as the local correlation between pairs of time series in the time and frequency domains, the wavelet coherence between pairs of signals was considered to be statistically significant ($\alpha = 0.05$)(() if its magnitude was greater than or equal to the 95th percentile of wavelet

coherence obtained from Monte Carlo simulations. Specifically, for each iteration of the Monte Carlo simulations, distinct pairs of surrogate time series were created using a first order autoregressive process whose parameters were estimated from the original time series. The wavelet coherence of the two surrogate time series was then computed at all locations in the time and frequency domains [41]. This process was repeated 999 times to generate a null distribution of wavelet coherence between surrogate time series and compute their 95th percentile [41]. These analyses were conducted using the biwavelet R package [43].

To characterize differences in environmental variability between study sites in Moorea and Taiwan, we compared the mean and the variance of pH and temperature time series. Monte Carlo randomizations were performed, as the data violated the assumptions (*e.g.*, normality) of more traditional parametric tests [44]. Specifically, for each metric, the null hypothesis that the values from both sites came from a single statistical population characterized by the same mean and variance was tested. For each randomization, values from the statistical population were randomly assigned to each site and then the between-site difference in the metric of interest was computed (*e.g.*, difference in mean or variance between sites). This generated a distribution of differences in the metric of interest under the null hypothesis. Then, the p-value was computed via a two-tailed test by determining the proportion of randomizations that yielded a difference in the metric of interest whose magnitude was greater than or equal to the one observed in the actual data. As with traditional parametric approaches, if the p-value generated via Monte Carlo randomizations is smaller than a predefined threshold (*i.e.*, $\alpha = 0.05$), then the null hypotheses can be rejected, and the difference between sites in the metric of interest is significant at the α level. In addition to this analysis testing the difference in environmental parameters across the distribution of pH, temperature, and depth values recorded, histograms were used to visualize the distribution of pH across percentiles. Monte Carlo randomizations were also performed to test the difference between environmental parameters of Moorea and Taiwan for each percentile. This analysis allowed us to determine whether the differences between sites based on the central tendency (mean) were also observed across the different percentiles of each environmental metric.

Results

The sites in Moorea and Taiwan had distinctly different profiles of seawater characteristics as assessed using summary statistics (Table 1). Firstly, use of SeaFETs to measure seawater pH required the collection of discrete water samples for data calibration. The seawater chemistry for the bottle sample in Moorea was: $pH_{total} = 8.026$; salinity = 35.65; $A_T = 2353$ μmol kg SW^{-1}. The seawater chemistry for the discrete bottle sample in Taiwan was: $pH_{total} = 8.006$, salinity = 33.43; $A_T = 2181$ μmol kg SW^{-1}. Mean seawater pH, temperature, Ω_{arag}, Ω_{calc}, and A_T were greater in Moorea than in Taiwan. However, mean pCO_2 was higher in Taiwan. The extreme values and amplitude of fluctuations of abiotic factors were also different between the two sites. Temperature, Ω_{arag}, and Ω_{calc} reached higher maxima in Moorea, but lower minima in Taiwan, with 3 times greater range of temperature in Taiwan. For seawater pH and pCO_2, the range was similar between sites, but Moorea had lower maxima and minima.

Dynamics of environmental pH

pH time series at both sites are characterized by strong and consistent daily fluctuations (Fig 1a and 1b). A portion of the Moorea pH time series was removed because these data failed to meet quality control standards. Wavelet analysis revealed areas of significant variability in pH at periods of ~24 hours that were consistent for Moorea but intermittent for Taiwan (Fig 1c and 1d). Throughout the entire time series, both Moorea and Taiwan exhibited intermittent high

Table 1. Summary of oceanographic conditions at fringing reefs in Moorea, French Polynesia and Taiwan in 2012.

Site	Summary Statistics	Temperature (°C)	Salinity	pH	A_T (μmol kg SW^{-1})	Ω_{arag}	Ω_{calc}	pCO_2 (μatm)
Moorea	*n*	8001	1	8001	1	8001	8001	8001
	Mean	28.76	35.65	7.989	2353	3.57	5.35	473
	SD	0.61	NA	0.038	NA	0.26	0.38	53
	Range	3.26	NA	0.233	NA	1.39	2.09	344
	Maximum	30.63	NA	8.069	NA	4.10	6.13	718
	Minimum	27.34	NA	7.836	NA	2.70	4.05	373
	25%	28.32	NA	7.966	NA	3.53	5.30	431
	75%	29.18	NA	8.019	NA	3.40	5.10	503
Taiwan	*n*	11012	11012	11012	1	11012	11012	11012
	Mean	27.74	33.12	7.974	2181	3.03	4.57	467
	SD	1.40	0.35	0.0315	NA	0.21	0.31	42
	Range	10.47	4.52	0.255	NA	1.67	2.42	337
	Maximum	30.01	34.56	8.105	NA	3.97	5.97	655
	Minimum	19.54	30.04	7.850	NA	2.30	3.55	318
	25%	27.14	32.96	7.953	NA	2.97	4.35	437
	75%	28.70	33.34	7.996	NA	3.18	4.80	494

pH (total scale) was measured by a SeaFET. Temperature was measured by duplicate thermistors in Moorea. Temperature and salinity were measured using a CT sensor in Taiwan. Salinity is expressed as a unitless quantity representing kg of dissolved solids per kg seawater. In Moorea, salinity was measured from a discrete water sample. A_T was measured from a discrete seawater sample at each site.

doi:10.1371/journal.pone.0121742.t001

wavelet power at lower periods (<16 hours; Fig 1c and 1d). In Moorea, the dark blue portion of the figure corresponds to the section of the pH time series that was excluded from the analysis. Integrating across time, the global wavelet power in Moorea had a much stronger peak in power at periods of ~24 hours because variability at that particular period persisted over the entire time series for Moorea but was only intermittent for Taiwan. Moorea also exhibited a smaller peak at periods of ~10-hours, which was driven primarily by significant variability at that period in the latter half of the pH time series (Fig 1e). Indeed, the scale-averaged wavelet power, which represents the time series of the total variance across all periods, showed prominent peaks in the latter half of the time series for Moorea due to the occurrence of this significant variability at lower periods (Fig 1f). However, the scale-averaged wavelet power for Taiwan was relatively low and exhibited less variability over time than that of Moorea (Fig 1f and 1g). Overall, the global wavelet power and scale-averaged wavelet power of Moorea were greater than their respective counterparts for Taiwan, indicating that Moorea was characterized by greater pH variability than Taiwan at all periods (Fig 1e through 1g).

When considering the entire distribution of pH values, the Taiwan site spent more time at lower pH (Fig 2a). When distributions of seawater pH were compared between Moorea and Taiwan, the Moorea time series contained more values of high seawater pH while the distribution in Taiwan was shifted lower, with more occurrences of low seawater pH (Fig 2a). Plotting the data as a distribution of pH over percentiles of the dataset, seawater pH in Moorea was greater than seawater pH in Taiwan for high percentiles but not low percentiles (Fig 2b). Monte Carlo randomizations were used to test the difference between pH in Moorea and Taiwan for each percentile of the pH distributions. The observed differences in pH between sites at all but one quantile were statistically significant (Fig 2c). The smallest pH values were significantly lower in Moorea than in Taiwan (Fig 2c), as suggested by the summary statistics (Table 1). However, seawater pH was significantly lower in Taiwan for the majority of the

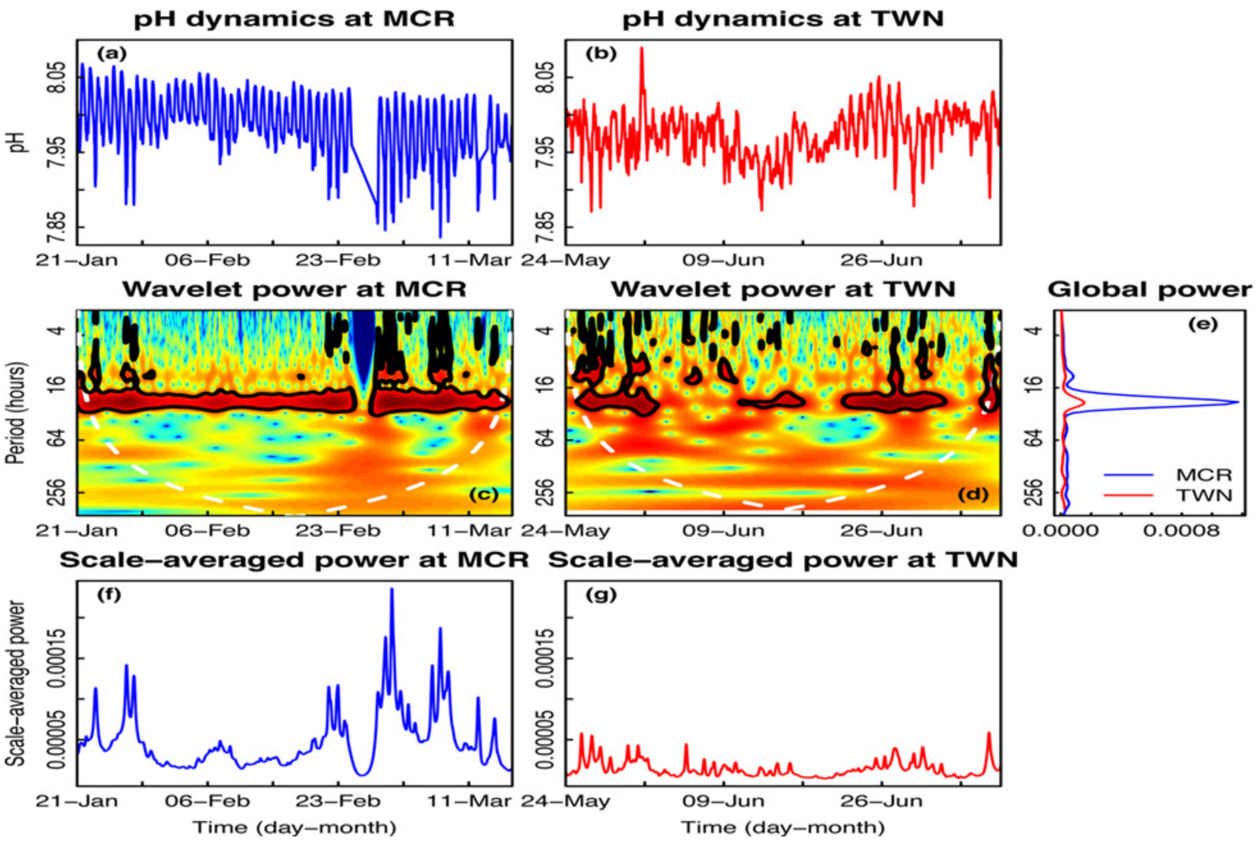

Fig 1. Time series and wavelet power analyses of seawater pH. Time series plots of seawater pH collected on a fringing reef in (a) Moorea [MCR], French Polynesia and (b) Taiwan [TWN]. Wavelet power quantifies the changes in relative contribution of each period in the pH signal to the overall variance of the signal for time series collected in (c) Moorea and (d) Taiwan. High power is represented in warm colors and low power is represented in cold colors. Black contours designate regions of significantly high temporal variation compared to a null model (red noise). The white line represents the cone of influence—values outside of this region are less reliable due to edge effects in the analysis. (e) Sites were compared using wavelet power integrated over time. Wavelet power was also integrated over period to describe changes in total variance over time for MCR (f) and TWN (g).

doi:10.1371/journal.pone.0121742.g001

distribution (Fig 2c). The non-parametric Kolmogorov-Smirnov test confirmed that there is a significant difference in the overall distribution of pH values between the sites ($p < 0.001$). Overall, the observed differences in mean and variance of pH were more extreme than those expected if the differences were drawn from a single statistical population, based on 999 Monte Carlo randomizations ($p < 0.001$ for both).

Dynamics of environmental temperature

Temperature time series at both sites were characterized by strong daily fluctuations, with some larger period depressions in temperature in Taiwan (Fig 3a and 3b). The plots of wavelet power show that temperature varied at different periods and the periodicity of the signal varied through time (Fig 3c and 3d). Throughout the entire time series for Moorea, high wavelet power was observed on a ~24-hour period, with some intermittent significant fluctuations occurring at lower periods (<16 hours; Fig 3c). In Taiwan, wavelet power at the 24-hour period was not consistently high, only significant during the second half of the time series (Fig 3d). During the second half of the time series in Taiwan, temperature also exhibited significant variability at lower periods (<16 hours; Fig 3d). However, during the first portion of the time

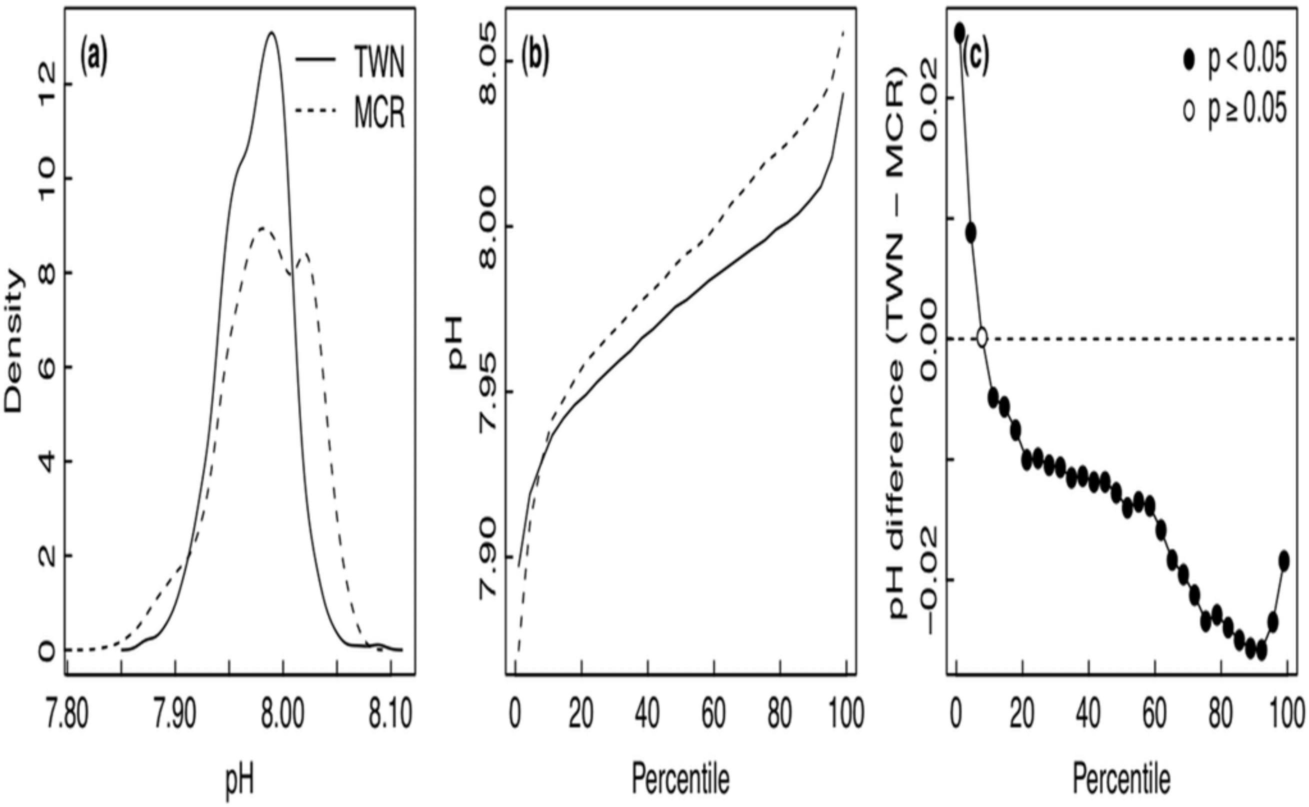

Fig 2. Comparisons of seawater pH between Moorea [MCR] and Taiwan [TWN]. (a) Histogram of the frequency densities of pH values observed during the deployments. (b) Distributions of frequencies of pH values during the deployments. (c) Differences in pH (TWN—MCR) for each percentile of the distribution of seawater pH, tested whether different from zero using Monte Carlo randomizations.

doi:10.1371/journal.pone.0121742.g002

series, there were no periods of significant variability (Fig 3d). The Taiwan dataset also contained a unique feature of strong power on a 2-week period (Fig 3d). Integrating across time, the global wavelet power spectrum in Moorea exhibited a single peak at periods of ~24-hours (Fig 3e). However, the global wavelet power spectrum for Taiwan was much greater than that of Moorea at all periods, indicating much stronger variation in temperature in Taiwan relative to Moorea over the length of the time series (Fig 3e). Global wavelet power for temperature exhibited peaks at 12-, 24- and 256-hour periods (Fig 3e). The scale- (or period-) averaged power was greater in Taiwan than in Moorea, with extreme peaks in the former occurring in early June and July (Fig 3f and 3g). Overall, the global wavelet power and scale-averaged wavelet power of Taiwan were greater than their respective counterparts for Moorea, indicating that Taiwan was characterized by greater temperature variability than Taiwan at all periods (Fig 3e through 3g).

The observed differences in the mean and variance of temperature were also significant between sites based on 999 Monte Carlo randomizations ($p < 0.001$ for both). When distributions of seawater temperature were compared between Moorea and Taiwan, the Moorea time series was concentrated around values of higher seawater temperature while the distribution in Taiwan had a tail with more occurrences of lower seawater temperature (Fig 4a). Plotting the data as a distribution of temperature over percentiles of the dataset, seawater temperature in Moorea was greater than seawater temperature in Taiwan for all percentiles (Fig 4b). Monte Carlo randomizations were used to test the difference between temperature in Moorea and

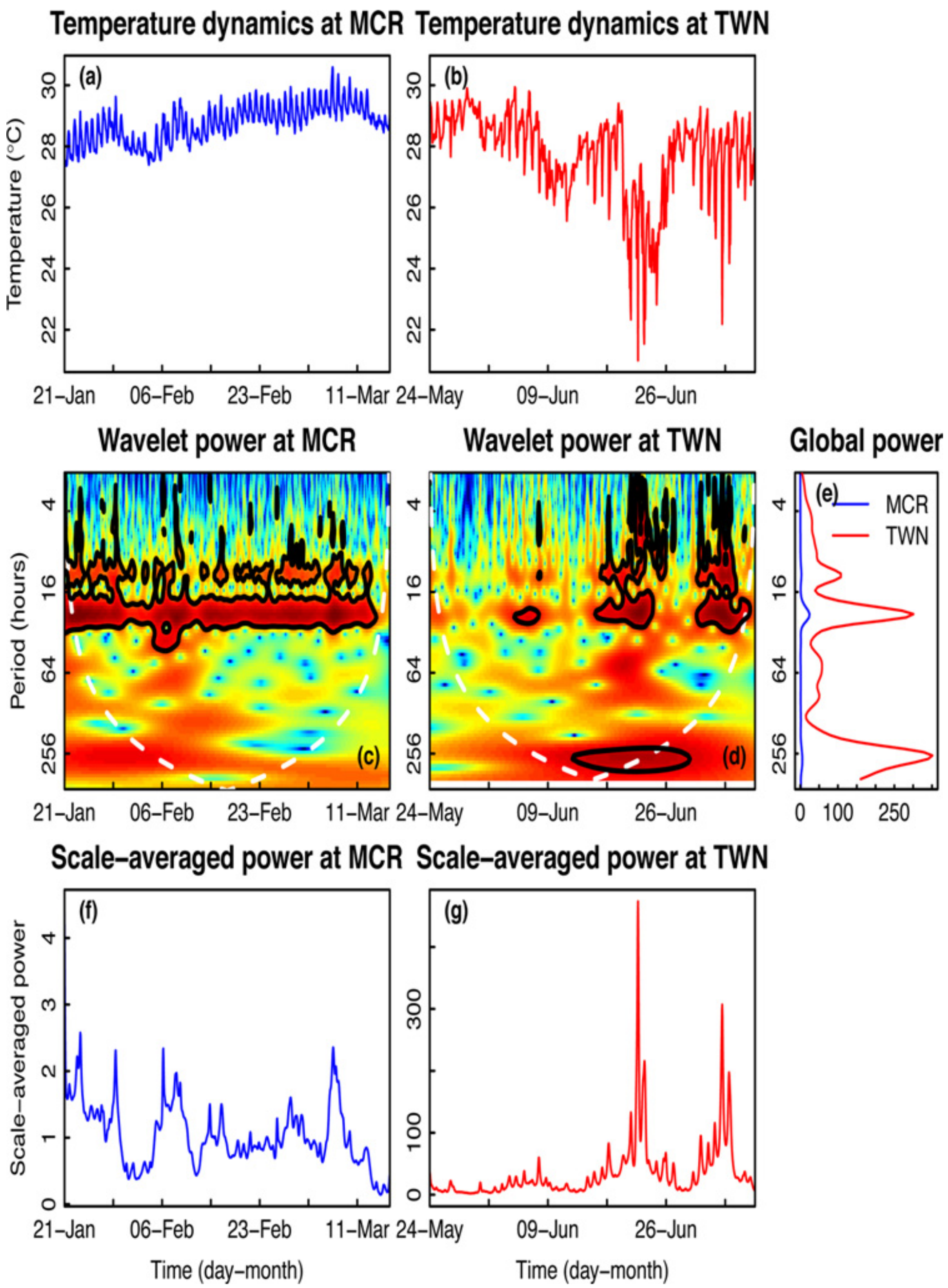

Fig 3. Time series and wavelet power analyses of seawater temperature. Time series plots of seawater temperature collected on a fringing reef in (a) Moorea [MCR], French Polynesia and (b) Taiwan [TWN]. Wavelet power quantifies the changes of relative contribution of each period in the temperature signal to the overall variance of the signal for time series collected in (c) MCR and (d) TWN. (e) Sites were compared using wavelet power integrated over time. Wavelet power was also integrated over period to describe changes in total variance over time for MCR (f) and TWN (g).

doi:10.1371/journal.pone.0121742.g003

Taiwan for each percentile of the temperature distributions. The observed differences in temperature between sites were statistically significant for all quantiles (Fig 4c). The most common and most rare temperature values were significantly higher in Moorea than in Taiwan (Fig 2c), as suggested by the summary statistics (Table 1).

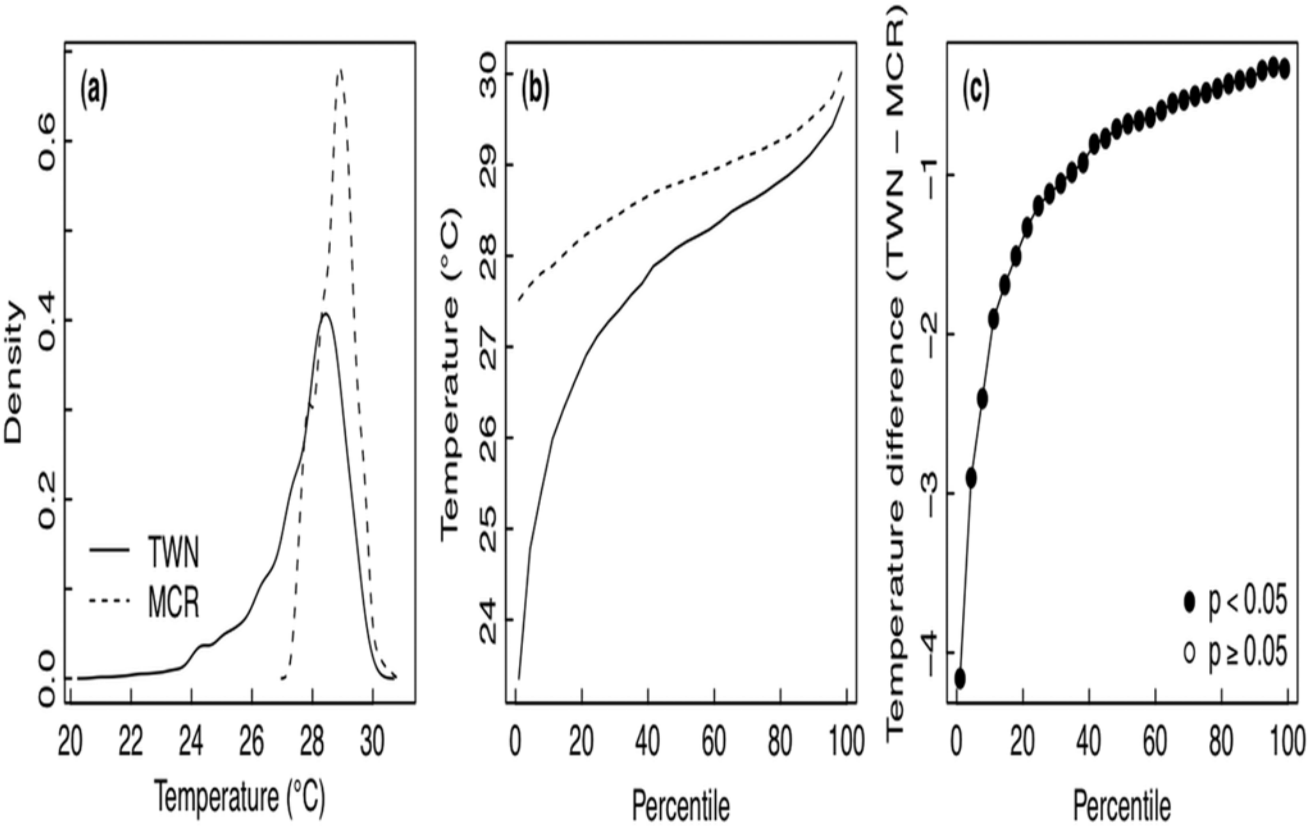

Fig 4. Comparison of seawater temperature between Moorea [MCR] and Taiwan [TWN]. (a) Histogram of the frequency densities of temperature values observed during the deployments. (b) Distributions of frequencies of temperature values during the deployments. (c) Differences in temperature (TWN—MCR) for each percentile of the distribution of seawater temperature, tested whether different from zero using Monte Carlo randomizations.

doi:10.1371/journal.pone.0121742.g004

Correlation between pH and temperature variability

The correlation between pH and temperature through time was determined for time series at each site using wavelet coherence. In Moorea, pH and temperature were significantly correlated through time on a ~24-hour period, with significant intermittent coherence at lower periods (<16 hours; Fig 5a). In Taiwan, a strong coherence between pH and temperature was sometimes present on a 24-hour period, but unlike Moorea, it was not consistent throughout the length of the time series. Lower periods also occasionally exhibited significant coherence in Taiwan (Fig 5b). As with any correlation study, it is important to note that strong coherence does not necessarily imply that one signal is driving the other or vice versa [40]. Both signals could have strong independent cycles of the same period.

Dynamics of environmental salinity

Salinity in Taiwan was characterized by strong daily fluctuations, with some larger period dips that coincided with larger decreases in temperature (Figs 3b and 6a). The distribution of salinity values was left skewed, with a longer tail of lower values (Fig 6b). The plot of wavelet power shows that salinity varied significantly at different periods over time (Fig 6c). Throughout the entire time series for Taiwan, wavelet power was intermittently high at a period of ~24 hours, with occasional significant fluctuations occurring at lower periods (<16 hours) and at 64-hour to two-week periods (Fig 6c). Integrating across time, the global power of salinity was

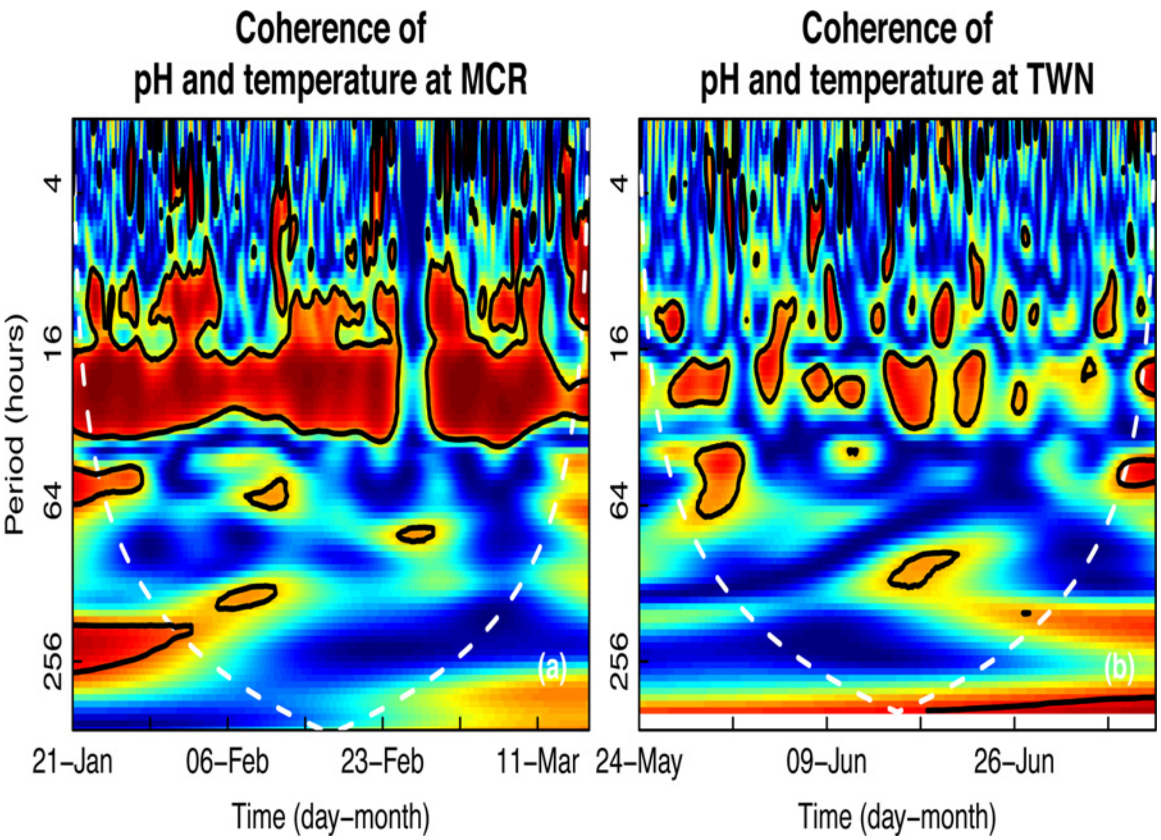

Fig 5. Correlation between pH and temperature fluctuations in seawater. Wavelet coherence, measuring the time-resolved correlation at each frequency or period for time series collected in (a) Moorea [MCR] and (b) Taiwan [TWN]. Warm colors indicate regions of high correlation, while cool colors indicate regions of low correlation. Black lines outline regions with significantly greater correlation.

doi:10.1371/journal.pone.0121742.g005

concentrated at the 24-hour and two-week periods (Fig 6d). Scale-averaged power was greatest for a discrete period in early June and elevated again around June 21 (Fig 6e). Wavelet coherence was performed in order to document the time- and frequency-resolved correlation between pH and salinity after partialling-out the effects of temperature. Once the effects of temperature were partialled-out, pH and salinity were not significantly correlated for the majority of the time-frequency domain (*i.e.*, abundance of dark blue color in Fig 6c). However, at intermittent times, the partial coherence between pH and salinity was significant around the 24-hour period (Fig 6f).

Dynamics of seawater depth

Depth time series at both sites are characterized by strong and consistent daily fluctuations, with some larger period oscillations (Fig 7a and 7b). Water depth oscillated with two minima and maxima per day at each location. However, in Moorea, each of the two oscillations had a consistent height, while in Taiwan, there was one large oscillation and one small oscillation per day (Fig 7a and 7b). Based on observations of the raw time series, the signals appeared to contain strong daily and lunar tidal signals. Throughout the entire time series for Moorea, high wavelet power was observed on a ~12-hour period, with no significant fluctuations at other periods (Fig 7c). In Taiwan, wavelet power at the 12-hour period was also consistently high, with evenly spaced high fluctuations at 24-hour periods (Fig 7d). Integrating across time, the global power in Moorea and Taiwan were elevated for 12- and 24-hour periods, although the average

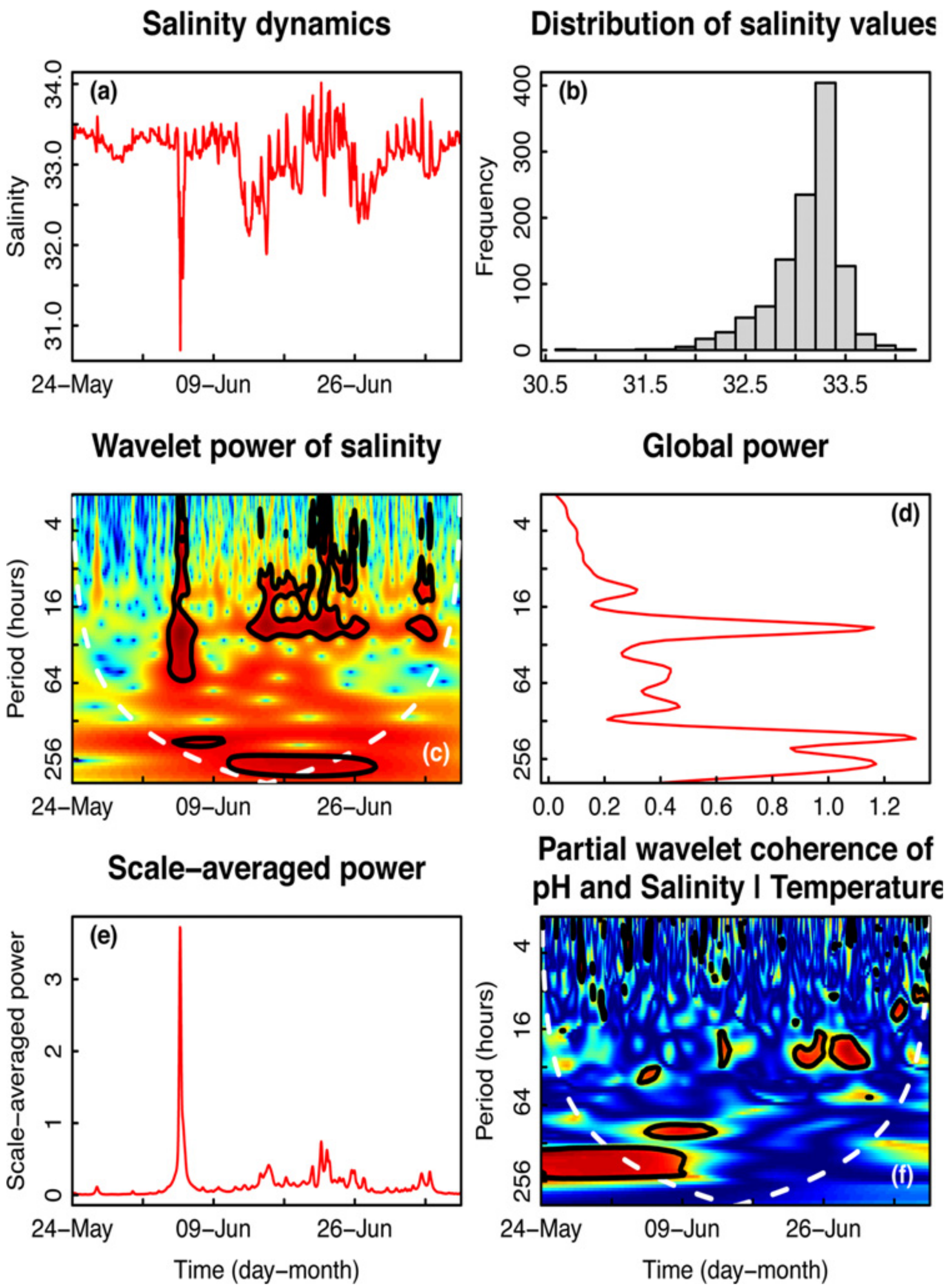

Fig 6. Time series and wavelet power analyses of seawater salinity in Taiwan. (a) Time series plot of seawater salinity collected on a fringing reef in Taiwan [TWN]. Salinity is expressed as a unitless quantity representing kg of dissolved solids per kg seawater. (b) Histogram of frequencies of salinity. (c) Wavelet power quantifies the changes of relative contribution of each period in the pH signal to the overall variance of the signal for time series collected in Taiwan. See Fig 1 legend for details. (d) Wavelet power was integrated over time to describe changes in power over frequency. (e) Wavelet power was also integrated over period to describe changes in total variance over time. (f) Partial wavelet coherences of pH, salinity, and temperature describe correlation between the three parameters across frequency and time domains. High coherence is represented in warm colors and low power is represented in cold colors. Black contours designate regions of significantly high temporal variation. The white line represents the cone of influence—values outside of this region are less reliable due to edge effects in the analysis.

doi:10.1371/journal.pone.0121742.g006

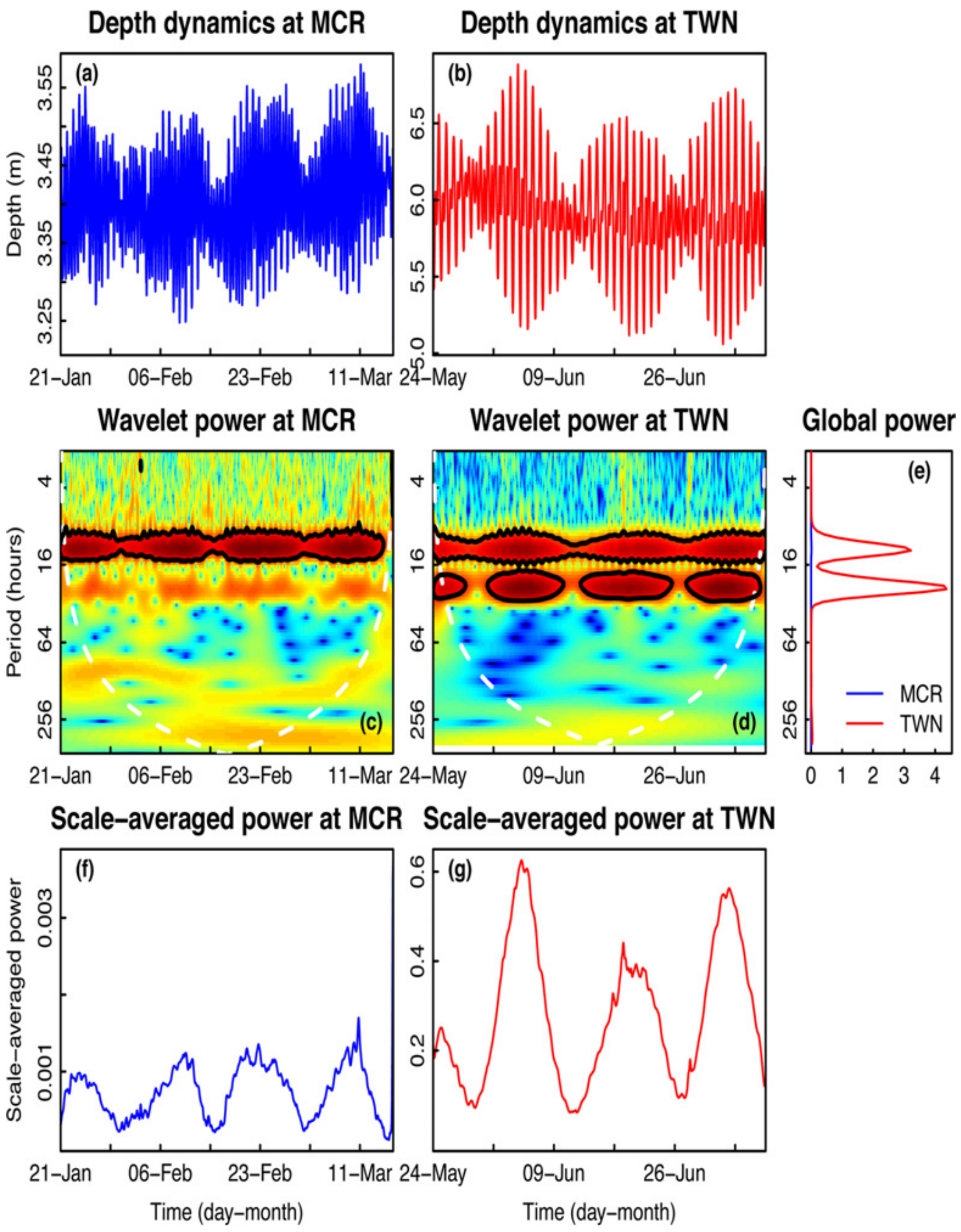

Fig 7. Time series and wavelet power analyses of seawater depth. Time series plots of seawater depth collected on a fringing reef in (a) Moorea [MCR], French Polynesia and (b) Taiwan [TWN]. (c) Distributions of frequencies of depth values during the deployments. Wavelet power quantifies the changes of relative contribution of each period in the depth signal to the overall variance of the signal for time series collected in (c) MCR and (d) TWN. (e) Sites were compared using wavelet power integrated over time. Wavelet power was also integrated over period to describe changes in total variance over time for MCR (f) and TWN (g).

doi:10.1371/journal.pone.0121742.g007

power of the Taiwan time series was much greater than that of Moorea at those periods (Fig 7e). Scale-averaged power peaked every two weeks in Moorea and in Taiwan, with greater amplitudes in Taiwan (Fig 7f and 7g).

When distributions of seawater depth were compared between Moorea and Taiwan, the Moorea time series contained a much narrower range of values, while the distribution in

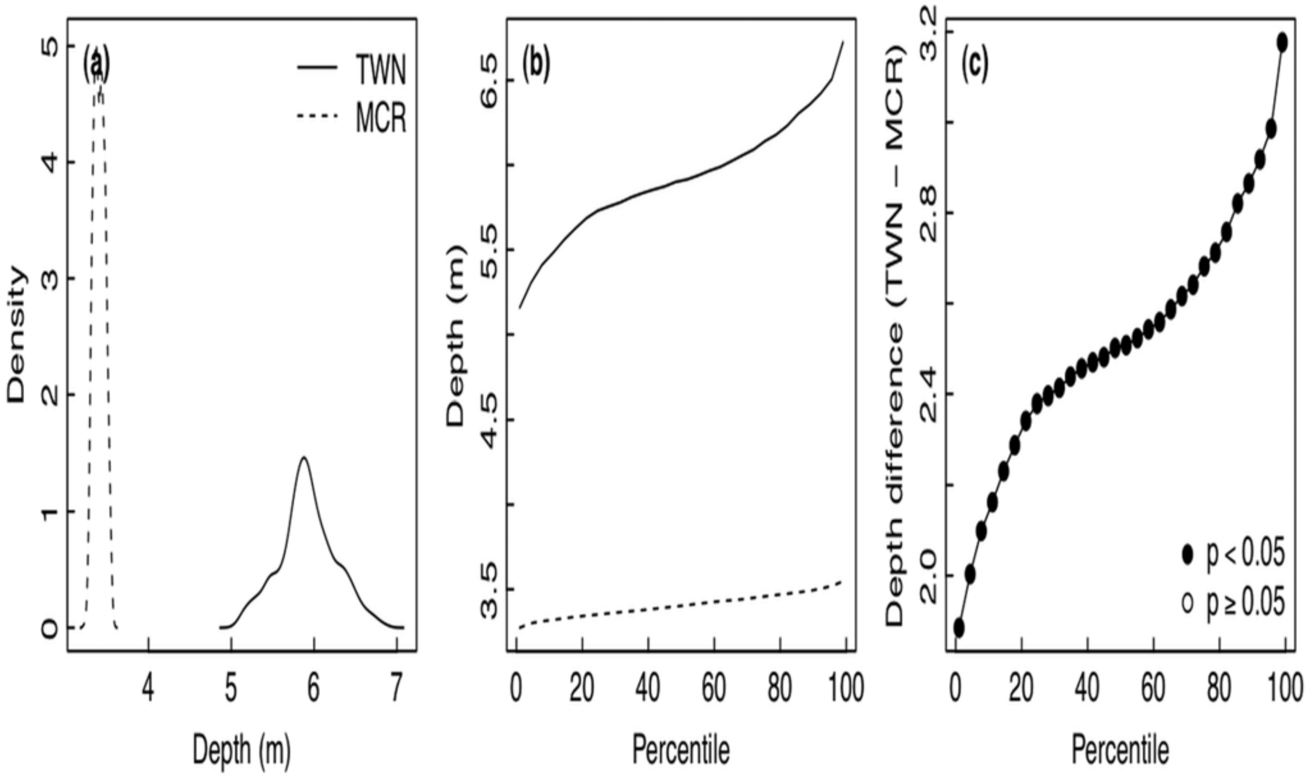

Fig 8. Comparison of seawater depth between Moorea [MCR] and Taiwan [TWN]. (a) Histogram of the frequency densities of depth values observed during the deployments. (b) Distributions of frequencies of depth values during the deployments. (c) Differences in depth (TWN—MCR) for each percentile of the distribution of seawater depth, tested whether different from zero using Monte Carlo randomizations.

doi:10.1371/journal.pone.0121742.g008

Taiwan was much wider (Fig 8a). Plotting the data as a distribution of depth over percentiles of the dataset, seawater depth in Taiwan was greater than in Moorea for all percentiles (Fig 8b). Monte Carlo randomizations were used to test the difference between temperature in Moorea and Taiwan for each percentile of the depth distributions. The observed differences in depth between sites were statistically significant for all quantiles (Fig 8c). The most common and most rare depth values were significantly higher in Taiwan than in Moorea (Fig 8c).

Discussion

In this study, we compared present-day environmental characteristics of the water masses bathing two fringing reefs, which span the biogeographic range of many Indo-Pacific coral species. Autonomous sensors generated datasets of pH, temperature, salinity, and depth that were analyzed to describe the abiotic conditions to which resident coral populations are potentially locally adapted or acclimatized, and further, to place the experimental treatments used in laboratory exposures in an ecologically-relevant context. We showed that coral populations experience complex patterns of spatiotemporal environmental variability across their biogeographical range, with different potential causal processes such as internal waves, upwelling, and typhoons leaving distinct temporal signatures that could only be resolved using advanced time series analysis. Theory suggests that such complex patterns of temporal environmental variability can lead to double jeopardy for coral populations (*i.e.*, threats from two sources) by reducing both their mean abundance and temporal stability, and thus increasing their risk of stochastic extinction across spatial scales.

Uncovering and explaining 'hidden' spatial heterogeneity in pH forcing

There are several reasons why pH and temperature regimes may differ between Moorea and Taiwan. First, although both time series were collected during the austral summer at each site, they did not overlap in time. Therefore, it is possible that the differences in environmental variability attributed to space may be due to temporal changes in the regimes of pH and temperature. In a small-scale study along a 32 m slope of a fringing reef at Coconut Island, Oahu, Guadayol and others [8] found that spatial changes in variability of abiotic parameters like pH and temperature exceeded temporal changes in the regime of these variables. The Hawaii study employed a spatio-temporal sampling design with more coverage than our study had with one set of sensors at each location. In comparison to the Hawaii study, our study encompassed a larger biogeographic spatial scale, where ratios between the variances of pH and temperature may not have been constant through time [8]. Our ~6-week deployments likely did not capture temporal changes in pH and temperature regimes across the austral summer seasons.

Different oceanographic features in Moorea and Taiwan likely contributed to the observed differences in pH and temperature regimes. In Nanwan Bay in Taiwan, upwelling induced by internal tides is known to cause large drops in temperature within a few hours, a phenomenon that is exacerbated when monsoon winds raise the thermocline in the bay [33,45]. During this study, Typhoon Mawar in early June and supertyphoon Guchol in late June may have caused upwelling in Nanwan Bay and subsequently drove the large-period depressions in seawater temperature observed at the Taiwan reef site (Fig 3b). Additionally, the flood and ebb eddies established near the study site in Nanwan Bay during spring tides have been reported to cause two temperature drops per day [33]. This phenomenon may account for the strong power of a ~2-week period observed in fluctuations of seawater temperature in Taiwan (Fig 3d). It was previously unknown whether the temperature-characterized upwelling in Nanwan Bay also caused changes in seawater pH. The results of this study suggest that fluctuations in temperature and pH in Taiwan at periods <10 hours are not strongly correlated (Fig 5b).

With respect to the site in French Polynesia, the fringing reef in Moorea was more protected due to its location inside a lagoon. Circulation patterns in Moorea are driven more by waves than by wind or tides [46]. In general, the lagoon is flushed as water comes over the barrier reef and flows out through the passes [46]. Because the Moorea site is more protected, fluctuations in pH and temperature may be less affected by oceanographic features and thus dominated by a diel period attributed to biological processes and the light regime.

Differences in weather patterns between Moorea and Taiwan could have contributed to their unique environmental regimes. Due to the two typhoons, the Taiwan site experienced ~1600 mm of rainfall during May-July, 2012 (Central Weather Bureau, Taiwan). In contrast, the island adjacent to Moorea (Tahiti) experienced ~1700 mm of rainfall during the entire year of 2012 (www.tutiempo.net), suggesting that heavy rainfall may have a more dramatic effect on the pH and temperature dynamics observed in Taiwan than in Moorea. In addition to inducing upwelling, the stormy weather in Taiwan could also have reduced seawater temperature due to decreased solar heating of the surface ocean. Interestingly, the coherence between pH and temperature was not strong during this time, so any upwelling enhanced by the typhoons likely did not alter the pH regime in Taiwan. Additionally, high rainfall during typhoons likely caused the observed broad decreases in salinity in Taiwan (Fig 6a).

Water depth plays an important role in the variability of pH and temperature. Mixing, turbulence, light intensity and quality, nutrient concentrations, and the influence of waves all vary by depth. Due to constraints in the specific deployment locations of the sensors, sensor depth differed between sites. Sensors were deployed at ~3 m in Moorea and at ~6 m in Taiwan. This difference may help explain why the 24-hour period in fluctuations of pH and temperature is

much stronger and more consistent in Moorea. The deeper depth in Taiwan likely dilutes the signal of photosynthesis and respiration within the overall variability of pH and temperature. Similarly, Guadayol *et al.* [8] also found that the importance of daily fluctuations within pH and temperature signals decreases with water depth. Water depth was also affected by semidiurnal tides present at both sites. Here, we show that changes in water depth associated with waves and tides are much more diverse in Taiwan than in Moorea (Fig 7c). The wide range of water depth may have weakened the power of the 24-hour period in the pH and temperature fluctuations in Taiwan (Figs 1d and 3d).

Biological implications of environmental fluctuations

The environmental data collected at the study sites approximated conditions experienced at the reef scale as well as conditions coral larvae may have encountered in the water column shortly after release. It is important to note that the measurements of pH and temperature presented here cannot account for gradients in pH and temperature on the meter scale [8] or within the boundary layer of the corals themselves [47]. At both sites, the mean pCO_2 value calculated (467–473 μatm) was higher than the annual global atmospheric mean for 2012 (393 μatm; Conway and Tans, NOAA/ESRL [www.esrl.noaa.gov/gmd/ccgg/trends]). Temperature stress may be more common in Moorea, where seawater temperature passed the local bleaching threshold (30°C; [48]) for several hours on three adjacent days in early March. In contrast, seawater temperature in Taiwan passed the local bleaching threshold (30°C; [49]) only once, for 6 minutes in May. As pH and temperature fluctuated on a variety of periods, aragonite saturation state at Moorea was on average above what is adequate for coral reefs (>3.5; [50,51]). However, Ω_{arag} fell as low as 2.70, a value at which coral accretion rates may be depressed [51]. Based on the summary statistics (Table 1), the study reef in Taiwan would be considered marginal and low-Ω_{arag}, with 75% of the time spent below commonly observed levels for coral reefs [50,51]. It is important to note the limitation of our ability to conclude much about Ω_{arag} at these sites as we collected limited datasets on salinity and total alkalinity.

pH and temperature time series recorded at Moorea and Taiwan differed slightly from conditions at other coral reefs. At Moorea, the mean pCO_2 value recorded (473 μatm) was higher than recorded in winter at this site in the previous year (374 μatm; [32]); lower wind stress in 2012 may have enhanced seawater retention in the lagoon, allowing the biological pCO_2 signal to increase average pCO_2 levels. In 2012 at Moorea, mean pH was lower and mean temperature was higher than in 2011 [32]. Mean pH variability reported here is similar to SeaFET pH time series data collected from coral reefs in the Northern Line Islands [4], but pH values at Moorea and Taiwan were lower than those reported for reef lagoons and reef flats on the Great Barrier Reef [11,16,52]. More acidic carbonate chemistry was documented in a lagoon in Okinawa and at Heron Island, where pCO_2 reached levels predicted in a worst-case scenario by the year 2100 [53–55]. Temperature in Taiwan was similar to that reported for Nanwan Bay previously [45].

Differing regimes of environmental conditions in Moorea and Taiwan may shape the ability of their coral populations to tolerate long-term environmental change. While a direct comparison of coral population data is not possible at this point, we can compare the results of several independent studies that examine the physiological responses of corals to changes in seawater pH and temperature. These studies focus on larvae of the reef-building coral, *Pocillopora damicornis*, whose post-release dispersal conditions are approximated by the oceanographic data we present. Conditions of OA have been shown to reduce respiration rates of *P. damicornis* larvae in Moorea [32], but various traits of larvae from populations in Taiwan (*e.g.*, respiration rates, maximum photosynthetic efficiency, mortality, protein content, *Symbiodinium* density) are often not affected by high pCO_2 [56–58]. Sensitivity to temperature is also complex—

temperature significantly affects larval physiology, but in Taiwan, the direction of this effect depends on the response variable measured, the day the larvae were released and length of exposure to experimental treatments [56–58]. A similar study conducted with the same species in Moorea revealed a positive effect of temperature on larval oxygen consumption that was more consistent across cohorts of larvae than reported by the comparable studies in Taiwan [32].

Moving beyond the mean to avoid the risk of double jeopardy

Overall, our time series analyses have revealed complex patterns of variation in pH at both sites. Mathematical theory based on Jensen's inequality suggests that such variability will potentially have a strong impact on the stability and persistence of coral populations if biological processes such as growth and recruitment are nonlinearly related to pH. Specifically, if these key coral population parameters are a decelerating function of pH, then increasing temporal variability in pH can lead to double jeopardy [59] by simultaneously reducing the mean and increasing the temporal variance of coral abundance. However, this double jeopardy risk can remain hidden if the temporal dynamics of pH are poorly documented or their impact on key organismal processes such as growth and mortality remain unknown. Hence our findings echo those of others [59,60] and suggest a more comprehensive operational definition of population vulnerability that integrates the effects of the mean and the temporal variance of key environmental stressors on both the mean and the temporal variance of abundance of potentially at-risk species. Our results further emphasize the importance of determining the functional relationship between environmental stressors and biological processes. Indeed, defining these functional responses is key for predicting the biological effects of temporal fluctuations in environmental conditions. Although this task may seem Sisyphean in complex and speciose ecosystems, the functional responses between biological and environmental processes are likely to be conserved across species, thus greatly reducing the logistical effort needed to adequately quantify these critical patterns.

A glimpse into the future: a space-for-time-substitution to address conservation goals

Regimes of pH and temperature differed between fringing reef sites in Moorea and Taiwan. Our in-depth comparisons showed that these differences persisted across comparisons of summary statistics, frequency distributions, and signal dynamics. It is likely that the relative importance of biological processes of the coral reef community (*i.e.*, photosynthesis, respiration, and calcification), oceanographic events, and weather at each site was distinct. As a result of the unique variability of pH and temperature between Moorea and Taiwan, there is potential that resident corals are locally adapted and seasonally acclimatized to the observed conditions [61]. Consequent population-specific physiological differences across the biogeographic species ranges may result in a range of responses to future changes in pH and temperature at these sites.

By documenting spatiotemporal heterogeneity in present-day environmental conditions, we can identify locations that are experiencing pH or temperature conditions that are not expected to become globally widespread for decades. For example, along the US West Coast, there are several coastal locations where undersaturated seawater seasonally shoals [62] or where pH is changing rapidly [63,64]. We can integrate this information using a space-for-time approach to predict and potentially mitigate the effects of climate change. Indeed, locations currently experiencing extreme temperature and pH conditions could serve as a source of adapted or acclimatized individuals that already possess the biological toolkit necessary to survive and reproduce in average future ocean conditions. Stakeholders can use this information to actively

manage populations of interest by transplanting these adapted or acclimatized individuals to locations that are predicted to experience harsher conditions in the future.

Supporting Information

S1 Text. Wavelet analysis. A summary of the methods used to determine how fluctuations in pH and temperature both varied and co-varied over time at sites in Moorea and Taiwan. (DOC)

Acknowledgments

The authors wish to acknowledge Dr. Gretchen E. Hofmann for her support of this project. Keith Seydel and Dr. Tung-Yung Fan supported the deployments of oceanographic sensors. Chris Gotschalk provided valuable mentorship in early data analyses.

Author Contributions

Conceived and designed the experiments: EBR. Performed the experiments: EBR. Analyzed the data: EBR TCG. Contributed reagents/materials/analysis tools: EBR TCG. Wrote the paper: EBR TCG.

References

1. Martz TR, Connery JG, Johnson KS, Road EW, Glen M (2010) Testing the Honeywell Durafet (R) for seawater pH applications. Limnol Oceanogr-Methods 8: 172–184.
2. Hofmann GE, Smith JE, Johnson KS, Send U, Levin LA, Micheli F, et al. (2011) High-frequency dynamics of ocean pH: a multi-ecosystem comparison. PLoS One 6: e28983. doi: 10.1371/journal.pone.0028983 PMID: 22205986
3. Frieder CA, Nam SH, Martz TR, Levin LA (2012) High temporal and spatial variability of dissolved oxygen and pH in a nearshore California kelp forest. Biogeosciences 9: 3917–3930.
4. Price NN, Martz TR, Brainard RE, Smith JE (2012) Diel variability in seawater pH relates to calcification and benthic community structure on coral reefs. PLoS One 7: e43843. doi: 10.1371/journal.pone.0043843 PMID: 22952785
5. Boatta F, D'Alessandro W, Gagliano AL, Liotta M, Milazzo M, Rodolfo-Metalpa R, et al. (2013) Geochemical survey of Levante Bay, Vulcano Island (Italy), a natural laboratory for the study of ocean acidification. Mar Pollut Bull 73: 485–494. doi: 10.1016/j.marpolbul.2013.01.029 PMID: 23465567
6. Yu PC, Matson PG, Martz TR, Hofmann GE (2011) The ocean acidification seascape and its relationship to the performance of calcifying marine invertebrates: Laboratory experiments on the development of urchin larvae framed by environmentally-relevant pCO_2/pH. J Exp Mar Biol Ecol 400: 288–295.
7. Hofmann GE, Blanchette CA, Rivest EB, Kapsenberg L (2013) Taking the pulse of marine ecosystems: The importance of coupling long-term physical and biological observations in the context of global change biology. Oceanography 26: 140–148.
8. Guadayol O, Silbiger NJ, Donahue MJ, Thomas FIM (2014) Patterns in temporal variability of temperature, oxygen and pH along an environmental gradient in a coral reef. PLoS One 9: e85213. doi: 10.1371/journal.pone.0085213 PMID: 24416364
9. Hofmann GE, Evans TG, Kelly MW, Padilla-Gamino JL, Blanchette CA, Washburn L, et al. (2014) Exploring local adaptation and the ocean acidification seascape—studies in the California Current Large Marine Ecosystem. Biogeosciences 11: 1053–1064.
10. Chierici M, Fransson A (2009) Calcium carbonate saturation in the surface water of the Arctic Ocean: undersaturation in freshwater influenced shelves. Biogeosciences 6: 2421–2431.
11. Gagliano M, McCormick MI, Moore JA, Depczynski M (2010) The basics of acidification: baseline variability of pH on Australian coral reefs. Mar Biol 157: 1849–1856.
12. Hall-Spencer JM, Rodolfo-Metalpa R, Martin S, Ransome E, Fine M, Turner SM, et al. (2008) Volcanic carbon dioxide vents show ecosystem effects of ocean acidification. Nature 454: 96–99. doi: 10.1038/nature07051 PMID: 18536730

13. Wootton JT, Pfister CA, Forester JD (2008) Dynamic patterns and ecological impacts of declining ocean pH in a high-resolution multi-year dataset. Proc Natl Acad Sci U S A 105: 18848–18853. doi: 10.1073/pnas.0810079105 PMID: 19033205

14. Yates KK, Dufore C, Smiley N, Jackson C, Halley RB (2007) Diurnal variation of oxygen and carbonate system parameters in Tampa Bay and Florida Bay. Mar Chem 104: 110–124.

15. Yates KK, Halley RB (2006) Diurnal variation in rates of calcification and carbonate sediment dissolution in Florida Bay. Estuaries Coasts 29: 24–39.

16. Albright R, Langdon C, Anthony KRN (2013) Dynamics of seawater carbonate chemistry, production, and calcification of a coral reef flat, central Great Barrier Reef. Biogeosciences 10: 6747–6758.

17. Shaw EC, McNeil BI, Tilbrook B (2012) Impacts of ocean acidification in naturally variable coral reef flat ecosystems. J Geophys Res 117: C03038.

18. Evans TG, Chan F, Menge BA, Hofmann GE (2013) Transcriptomic responses to ocean acidification in larval sea urchins from a naturally variable pH environment. Mol Ecol 22: 1609–1625. doi: 10.1111/mec.12188 PMID: 23317456

19. Kelly MW, Padilla-Gamiño JL, Hofmann GE (2013) Natural variation and the capacity to adapt to ocean acidification in the keystone sea urchin *Strongylocentrotus purpuratus*. Glob Change Biol 19: 2536–2546. doi: 10.1111/gcb.12251 PMID: 23661315

20. Padilla-Gamiño JL, Kelly MW, Evans TG, Hofmann GE (2013) Temperature and CO_2 additively regulate physiology, morphology and genomic responses of larval sea urchins, *Strongylocentrotus purpuratus*. Proc R Soc B-Biol Sci 280: 20130155. doi: 10.1098/rspb.2013.0155 PMID: 23536595

21. Frieder CA (2014) Present-day nearshore pH differentially depresses fertilization in congeneric sea urchins. Biol Bull 226: 1–7. PMID: 24648202

22. Hauri C, Gruber N, Plattner G-K, Alin S, Feely RA, Hales B, et al. (2009) Ocean acidification in the California Current System. Oceanography 22: 60–71.

23. Fabricius KE, Langdon C, Uthicke S, Humphrey C, Noonan S, De'ath G, et al. (2011) Losers and winners in coral reefs acclimatized to elevated carbon dioxide concentrations. Nat Clim Change 1: 165–169.

24. Pandolfi JM, Connolly SR, Marshall DJ, Cohen AL (2011) Projecting coral reef futures under global warming and ocean acidification. Science 333: 418–422. doi: 10.1126/science.1204794 PMID: 21778392

25. Waldbusser GG, Voigt EP, Bergschneider H, Green MA, Newell RIE (2011) Biocalcification in the eastern oyster (*Crassostrea virginica*) in relation to long-term trends in Chesapeake Bay pH. Estuaries Coasts 34: 221–231.

26. Kelly MW, Hofmann GE (2012) Adaptation and the physiology of ocean acidification. Funct Ecol 27: 980–990.

27. Sanford E, Kelly MW (2011) Local adaptation in marine invertebrates. Annu Rev Mar Sci 3: 509–535. PMID: 21329215

28. Eliason EJ, Clark TD, Hague MJ, Hanson LM, Gallagher ZS, Jeffries KM, et al. (2011) Differences in thermal tolerance among sockeye salmon populations. Science 332: 109–112. doi: 10.1126/science.1199158 PMID: 21454790

29. Ruel JJ, Ayres MP (1999) Jensen's inequality predicts effects of environmental variation. Trends Ecol Evol 14: 361–366. PMID: 10441312

30. Shaw EC, Munday PL, McNeil BI (2013) The role of CO_2 variability and exposure time for biological impacts of ocean acidification. Geophys Res Lett 40: 4685–4688.

31. Gaitán-Espitia JD, Hancock JR, Padilla-Gamiño JL, Rivest EB, Blanchette CA, Reed DC, et al. (2014) Interactive effects of elevated temperature and pCO_2 on early-life-history stages of the giant kelp *Macrocystis pyrifera*. J Exp Mar Biol Ecol 457: 51–58.

32. Rivest EB, Hofmann GE (2014) Responses of the metabolism of the larvae of *Pocillopora damicornis* to ocean acidification and warming. PLoS One 9: e96172. doi: 10.1371/journal.pone.0096172 PMID: 24769774

33. Lee H-J, Chao S-Y, Fan K-L, Kuo T-Y (1999) Tide-induced eddies and upwelling in a semi-enclosed basin: Nan Wan. Estuar Coast Shelf Sci 49: 775–787.

34. Lee K, Tong LT, Millero FJ, Sabine CL, Dickson AG, Goyet C, et al. (2006) Global relationships of total alkalinity with salinity and temperature in surface waters of the world's oceans. Geophys Res Lett 33: L19605. PMID: 19122778

35. Dickson A, Sabine C, Christian J (2007) Guide to best practices for ocean CO_2 measurements. Sidney, British Columbia.

36. Robbins LL, Hansen ME, Kleypas JA, Meylan SC (2010) CO2calc—a user-friendly seawater carbon calculator for Windows, Mac OS X and iOS (iPhone). US Geological Survey Open-File Report 2010–1280.

37. Mehrbach C, Culberso CH, Hawley JE, Pytkowic RM (1973) Measurement of apparent dissociation constants of carbonic acid in seawater at atmospheric pressure. Limnol Oceanogr 18: 897–907.

38. Dickson AG, Millero FJ (1987) A comparison of the equilibrium constants for the dissociation of carbonic acid in seawater media. Deep Sea Res Part Oceanogr Res Pap 34: 1733–1743.

39. Torrence C, Compo GP (1998) A practical guide to wavelet analysis. Bull Am Meteorol Soc 79: 61–78.

40. Cazelles B, Chavez M, Berteaux D, Menard F, Vik JO, Jenouvrier S, et al. (2008) Wavelet analysis of ecological time series. Oecologia 156: 287–304. doi: 10.1007/s00442-008-0993-2 PMID: 18322705

41. Grinsted A, Moore JC, Jevrejeva S (2004) Application of the cross wavelet transform and wavelet coherence to geophysical time series. Nonlinear Process Geophys 11: 561–566.

42. Torrence C, Webster PJ (1998) The annual cycle of persistence in the El Niño/Southern Oscillation. Q J R Meteorol Soc 124: 1985–2004.

43. Gouhier T (2014) biwavelet: Conduct univariate and bivariate wavelet analyses. Available: http://github.com/tgouhier/biwavelet.

44. Manly BFJ (2006) Randomization, bootstrap and Monte Carlo methods in biology. Third. Chapman & Hall.

45. Jan S, Chen C-TA (2009) Potential biogeochemical effects from vigorous internal tides generated in Luzon Strait: A case study at the southernmost coast of Taiwan. J Geophys Res-Oceans 114.

46. Hench JL, Leichter JJ, Monismith SG (2008) Episodic circulation and exchange in a wave-driven coral reef and lagoon system. Limnol Oceanogr 53: 2681–2694.

47. Shashar N, Cohen Y, Loya Y (1993) Extreme diel fluctuations of oxygen in diffusive boundary layers surrounding stony corals. Biol Bull 185:455–461.

48. Gleason MG (1993) Effects of disturbance on coral communities: bleaching in Moorea, French Polynesia. Coral Reefs 12: 193–201.

49. Wilkinson C (1998) The 1997–1998 mass bleaching event around the world. In: Wilkinson CR, editor. Status of coral reefs of the world: 1998. Townsville, Australia: Australian Institute of Marine Science. pp. 15–38.

50. Guinotte JM, Buddemeier RW, Kleypas JA (2003) Future coral reef habitat marginality: temporal and spatial effects of climate change in the Pacific basin. Coral Reefs 22: 551–558.

51. Kleypas JA, McManus JW, Menez LAB (1999) Environmental limits to coral reef development: where do we draw the line? Integr Comp Biol 39: 146–159.

52. Shaw EC, McNeil BI (2014) Seasonal variability in carbonate chemistry and air–sea CO_2 fluxes in the southern Great Barrier Reef. Mar Chem 158: 49–58.

53. Ohde S, van Woesik R (1999) Carbon dioxide flux and metabolic processes of a coral reef, Okinawa. Bull Mar Sci 65: 559–576.

54. Santos IR, Glud RN, Maher D, Erler D, Eyre BD (2011) Diel coral reef acidification driven by porewater advection in permeable carbonate sands, Heron Island, Great Barrier Reef. Geophys Res Lett 38: L03604.

55. IPCC (2013) Summary for Policymakers. In: Stocker TF, Qin D, Plattner G-K, Tignor M, Allen SK, Boschung J, et al., editors. Climate Change 2013: The Physical Science Basis. Contribution of Working Group I to the Fifth Assessment Report of the Intergovernmental Panel on Climate Change. Cambridge, United Kingdom and New York, NY USA: Cambridge University Press.

56. Cumbo VR, Edmunds PJ, Wall CB, Fan TY (2013) Brooded coral larvae differ in their response to high temperature and elevated pCO_2 depending on the day of release. Mar Biol 160: 2903–2917.

57. Cumbo VR, Fan TY, Edmunds PJ (2013) Effects of exposure duration on the response of *Pocillopora damicornis* larvae to elevated temperature and high pCO_2. J Exp Mar Biol Ecol 439: 100–107.

58. Putnam HM, Mayfield AB, Fan TY, Chen CS, Gates RD (2013) The physiological and molecular responses of larvae from the reef-building coral *Pocillopora damicornis* exposed to near-future increases in temperature and pCO_2. Mar Biol 160: 2157–2173.

59. Gouhier TC, Guichard F, Menge BA (2013) Designing effective reserve networks for nonequilibrium metacommunities. Ecol Appl 23: 1488–1503. PMID: 24147418

60. Anderson CNK, Hsieh C, Sandin SA, Hewitt R, Hollowed A, Beddington J, et al. (2008) Why fishing magnifies fluctuations in fish abundance. Nature 452: 835–839. doi: 10.1038/nature06851 PMID: 18421346

61. Palumbi SR, Barshis DJ, Traylor-Knowles N, Bay RA (2014) Mechanisms of reef coral resistance to future climate change. Science: published online ahead of print.

62. Feely RA, Sabine CL, Hernandez-Ayon JM, Ianson D, Hales B (2008) Evidence for upwelling of corrosive "acidified" water onto the continental shelf. Science 320: 1490–1492. doi: 10.1126/science.1155676 PMID: 18497259

63. Wootton JT, Pfister CA (2012) Carbon system measurements and potential climatic drivers at a site of rapidly declining ocean pH. PloS One 7: e53396. doi: 10.1371/journal.pone.0053396 PMID: 23285290

64. Harris KE, DeGrandpre MD, Hales B (2013) Aragonite saturation state dynamics in a coastal upwelling zone. Geophys Res Lett 40: 2720–2725.
